# The Use and Reprocessing of Endodontic Files in Saudi Arabia: A Cross-Sectional Study

**DOI:** 10.7759/cureus.54427

**Published:** 2024-02-18

**Authors:** Majed Almalki, Waad Khayat

**Affiliations:** 1 Department of Restorative Dentistry, College of Dentistry, Umm Al-Qura University, Makkah, SAU

**Keywords:** sterilization, patient’s safety, infection control, endodontic file, dental infection

## Abstract

Objectives

This study aimed to assess the awareness, implementation, and compliance with the infection control policy recommended by the Saudi Ministry of Health (MOH) for the use and reprocessing of endodontic files and to examine the association between compliance and gender, experience, clinical ranks, and workplace sector.

Methods

This was a cross-sectional study conducted using a validated self-administered electronic questionnaire created and distributed to dentists who perform endodontic treatment in Saudi Arabia. Data were collected between June and July 2023. Descriptive statistics were reported as counts and percentages. Comparisons among the demographic groups were done using the Kruskal-Wallis and Mann-Whitney tests.

Results

A total of 402 dentists completed the survey. The results showed that 76.1% (n=306) of respondents claimed that they were aware of the infection control policy recommended by the Saudi MOH for the use and reprocessing of endodontic files in dental clinics. Only 13.2% (n=53) of dentists used single-use endodontic files, and 36.6% (n=147) did not use an endodontic box in their dental clinics. The most commonly reported method of tracing the number of uses of endodontic files was to write the ID of the patient or the number of uses on the sterilization pouch as reported by 37.6% of participants (n=151). The average compliance score percentage was 63.5 ± 16.7. Most of the respondents showed moderate to high levels of compliance (51.7% (n=208) and 42.0% (n=169) of dentists, respectively). Dentists with less than 5 years of experience showed significantly less compliance than dentists with more than 10 years of experience (p = 0.005). Gender, clinical rank, and workplace sector were not significantly associated with the extent of adhering to the evaluated infection control policy.

Conclusions

Our findings indicate a relatively high level of compliance with the Saudi MOH policy of using and reprocessing endodontic files. However, critical measures such as the single-use of endodontic files, sterilizing new endodontic files, and using the sterilized endodontic box for each patient need improvement. Hence, this study recommends enhancing awareness through continuous education and training.

## Introduction

Recently, the ethical and legal aspects of dental practice have received great attention worldwide. Compliance with infection control measures is one of the paramount ethical obligations to protect patients and dental personnel. Dental patients have a relatively high risk of infection. Thus, preventing and controlling the spread of infectious diseases is a critical concern in dentistry. Several standard precautions are recommended to control infection transmission in the dental setting [1-3]. In endodontics, root canal treatment is performed through multiple phases, including cleaning and shaping the canals using hand and rotary endodontic files. These files are subject to environmental and biological contamination. Thus, reprocessing and reusing them for different patients have raised concerns about the efficiency of the current infection control procedures [1,4].

Reprocessing and reusing endodontic files for multiple patients has been a common practice for years. However, several recent studies have demonstrated crucial issues, such as inadequate cleanliness, infectious disease transmission, and unreliability of the current decontamination method [5-9]. Therefore, healthcare authorities in the United Kingdom and several companies have recommended using endodontic files as single-use instruments [10]. Healthcare systems have developed guidelines and regulations to provide high-quality standards governed by ethics and laws to ensure safe and effective dental practice. The Ministry of Health (MOH) in Saudi Arabia published two editions of the Manual of Infection Prevention and Control in Dental Settings. The last edition presented specific infection control policies for each procedure in dentistry, including endodontics [3].

To the best of our knowledge, no previously published studies have investigated the awareness, implementation, and compliance with the infection control policy recommended by the Saudi MOH for the use and reprocessing of endodontic files. The findings of this study provide valuable insight into the extent to which dentists adhere to the infection control policy published by the Saudi MOH for the use and reprocessing of endodontic instruments in endodontic treatment in Saudi dental healthcare centers. Furthermore, the findings help identify areas of concern for further planning of efficient infection control training (if needed) to meet the quality requirements and accreditation standards of effective and safe patient-centered modern dental practice because failure to adhere to policies and regulations may cause practitioners to face legal issues and disciplinary actions.

Therefore, this study aimed to provide descriptive statistics on the common practice of using, reprocessing, and cleaning endodontic files in Saudi Arabia, assess the awareness, implementation, and compliance with the infection control policy recommended by the Saudi MOH for the use and reprocessing of endodontic files, and examine the association between compliance with the infection control policy and gender, experience, clinical ranks, and workplace sector.

## Materials and methods

This study was approved by the Institutional Review Board of Umm Al-Qura University (Approval number: HAPO-02-K-012-2023-02-1484). Participants were informed about the study purpose, the estimated time required to answer the questionnaire, and the anonymity, privacy, and confidentiality of data before participation. Consent to participate in the study was obtained from the participants before answering the electronic questionnaire by selecting and confirming their participation.

This was a cross-sectional study using a validated self-administered electronic questionnaire. The questionnaire was developed based on the latest edition of the Manual of Infection Prevention and Control in Dental Settings published by the Saudi MOH and a previously reported study in Germany. It was created electronically in English using Microsoft Forms (Microsoft 365) [3,11]. A preliminary pilot study was conducted to assess the reliability and validity of the questionnaire. The content validity was assessed by seven experts to evaluate the relevance and clarity of the questionnaire items and to modify the questionnaire accordingly. The questionnaire was then sent to 30 independent participants to test internal consistency and construct validity. Reliability was assessed using Cronbach’s alpha, which showed satisfactory reliability (Cronbach’s alpha = 0.72). Construct validity was examined using Pearson’s correlation coefficient for each item in correlation with the total score of all items. Values greater than the critical value of 0.349 at a 95% confidence level indicated valid items. All items were validated.

A link to the questionnaire was generated in 2023 and distributed to dentists through different social media platforms (WhatsApp, X, and Facebook groups) to collect data for two months (June and July). The snowball sampling technique was used by asking participants to share the questionnaire link with their colleagues, thereby increasing the pool of potential respondents. The inclusion criteria included dentists who performed endodontic treatment in the academic, military, governmental, or private dental healthcare sectors in Saudi Arabia. The exclusion criteria included endodontists who did not work in Saudi Arabia, undergraduate students, and general dentists who did not perform endodontic treatment.

The questionnaire included various sections. The first section included close-ended questions to determine eligibility to participate in the study. Participants who did not meet the inclusion criteria received a thank-you note and exited the questionnaire. Respondents who met the inclusion criteria were transferred to the next section to collect information about participants’ gender, workplace, clinical rank, and years of experience. The third section was about the awareness and implementation of the recommended infection control policy for the use of endodontic files, the use of endodontic box, the frequency of using endodontic files, and methods of tracing the number of using them for the same patients. The last section included questions about the sterilization of new endodontic files before first use, sterilization of the unused files in the endodontic box between patients, the removal of the rubber stopper before sterilization, the personnel responsible for cleaning and sterilizing endodontic instruments, and the manual and mechanical process of cleaning. Only completed questionnaires from eligible participants were submitted for analysis.

Compliance with the recommended policy was evaluated using a set of eight questions answered on a 3-point Likert scale, with 2 indicating always, 1 indicating sometimes, and 0 indicating never. The total compliance score for each participant was calculated as the sum of the scores of the eight items to assess the extent of adherence to the protocol. The maximum compliance score was 16. A total compliance score of 0 indicated no compliance, 1-5 indicated low compliance, 6-10 indicated moderate compliance, 11-15 indicated high compliance, and 16 indicated full compliance. The compliance score (%) was calculated and compared between the demographic groups.

The sample size was calculated based on the number of healthcare workers in Saudi Arabia (2018-2027) using the latest statistics published by the Saudi Commission of Health Specialties [12]. A sample size of 361 was needed to achieve a 95% confidence level and 5% margin of error for a population size of 5829 (including licensed general dentists, postgraduate residents in the Saudi Board Endodontics Residency Program, and classified endodontists in Saudi Arabia). Data entry and analysis were performed using Excel (Microsoft Office 365) and SPSS software (IBM SPSS Statistics for Mac, Version 28.0.1.0, IBM Corp., Armonk, NY, USA). Descriptive statistics were reported as counts and percentages. Median values were compared among the demographic groups using the Kruskal-Wallis and Mann-Whitney tests. Pairwise comparison tests were performed to determine which groups were significantly different from each other when applicable. A p-value < 0.05 indicated statistical significance. Bonferroni correction was applied for multiple comparisons.

## Results

A total of 402 respondents were included in the study. Table 1 shows the gender, experience, workplace, and clinical rank of the participants. A total of 306 participants (76.1%) reported that they were aware of the infection control policy recommended by the Saudi MOH for the use and reprocessing of endodontic files in dental clinics, and 286 participants (71.2%) reported that they followed the Manual of Infection Prevention and Control in Dental Settings, whereas others reported that they followed different policies, or were not sure which policy was applied in their workplaces.

**Table 1:** Distribution of participants based on variables of interest.

Variable of interest		n	%
Gender	Male	214	53.2
	Female	188	46.8
Experience	<5 years	172	42.8
	5–10 years	119	29.6
	>10 years	111	27.6
Workplace sector	Academic healthcare	41	10.2
	Governmental healthcare	117	29.1
	Military healthcare	96	23.9
	Private healthcare	148	36.8
Clinical rank	General dentist	211	52.5
	Postgraduate resident	54	13.4
	Specialist/consultant	137	34.1

Only 53 participants (13.2%) reported that they used single-use endodontic files. The study showed that 349 participants (86.8%) reported that they reused files; among them, 235 dentists (58.5%) used each file 2-4 times on average, 43 dentists (10.7%) used each file 5-7 times on average, and 71 dentists (17.7%) continued using each file until distortion was observed. The most reported method of tracing the number of uses of endodontic files was to write the ID of the patient or the number of uses on the sterilization pouch as reported by 151 participants (37.6%), followed by marking the number of uses on the rubber stopper as reported by 141 participants (35.1%). Other methods reported by 57 participants (14.1%) included recording marks on the files using permanent markers or storing files in different locations/drawers based on the number of uses. Furthermore, 147 dentists (36.5%) do not use an endodontic box in their dental clinics.

The process of cleaning, disinfecting, and sterilizing endodontic files was more commonly performed by dental assistants, as reported by 248 dentists (61.7%), than by specialized auxiliary infection control personnel, as reported by 154 dentists (38.3%). Mechanical disinfection of endodontic files using a thermal washer disinfector was more frequently used, as reported by 249 participants (61.9%), than manual disinfection with/without an ultrasonic bath, as reported by 126 participants (31.3%).

Table 2 shows descriptive data (frequency and percentage) on the use and reprocessing of endodontic files according to the policy recommended by the Saudi MOH. The mean compliance score percentage was 63.5% ± 16.7%. Most of the respondents showed moderate to high levels of compliance (51.7% (n=208) and 42.0% (n=169) of dentists, respectively). Table 3 shows the evaluation of adherence to the recommended policy (represented by the compliance score) and the comparison between the various demographic groups. The Kruskal-Wallis test revealed a statistically significant association between the extent of adherence to the recommended policy and clinical rank (p = 0.04). However, none of the post-hoc tests using the Bonferroni correction for multiple comparisons revealed a statistically significant association. Furthermore, dentists with a limited experience of less than 5 years showed significantly less compliance than dentists with more than 10 years of experience (p = 0.005).

**Table 2:** Data (frequency and percentage) on the use and reprocessing of endodontic files.

Item	Always n (%)	Sometimes n (%)	Never n (%)
I use each barbed broach only once and discard it after a single use	334 (83.0)	34 (8.5)	34 (8.5)
I follow the manufacturer’s instructions for decontamination and disposal of endodontic files	240 (59.7)	140 (34.8)	22 (5.5)
I wipe reusable endodontic files with sodium hypochlorite after each insertion into the root canal	147 (36.6)	146 (36.3)	109 (27.1)
I reuse the reusable endodontic files only on the same patient during multivisit root canal treatment	163 (40.5)	182 (45.3)	57 (14.2)
I do not use endo boxes or use each endo box for only one patient during endodontic treatment	208 (51.7)	147 (36.6)	47 (11.7)
I resterilize all the unused contents of the endo box/set before using them for multiple patients	327 (81.3)	21 (5.3)	54 (13.4)
I disinfect/sterilize new endodontic files before the first use	136 (33.8)	79 (19.7)	187 (46.5)
I remove the rubber stopper before sterilizing the reusable endodontic files	86 (21.4)	61 (15.2)	255 (63.4)

**Table 3:** Evaluation of the practice and adherence to the infection control policy for the use and reprocessing of endodontic files (compliance score) among the demographic groups. *p-values were calculated using the Kruskal–Wallis and Mann–Whitney tests. Different superscript letters indicate statistically significant differences at p < 0.05.

		Score	Compliance score %		
Variable of interest		Mean (SD)	Mean (SD)	Median (IQR)	p-value*
Gender	Male	10.3 (2.9)	64.1 (17.8)	62.5 (25.0)	0.49
	Female	10.1 (2.4)	62.9 (15.3)	62.5 (25.0)	
Experience	<5 years	9.8 (2.5)	61.0 (15.6)	62.5 (23.4) a	0.01
	5–10 years	10.4 (2.7)	65.0 (17.0)	62.5 (18.8) a, b	
	>10 years	10.6 (2.8)	66.0 (17.4)	68.8 (18.8) b	
Workplace sector	Academic healthcare	10.1 (2.5)	63.3 (15.6)	62.5 (12.5)	0.19
	Governmental healthcare	9.9 (2.5)	61.5 (15.5)	62.5 (25.0)	
	Military healthcare	10.1 (2.1)	63.1 (13.3)	62.5 (17.8)	
	Private healthcare	10.5 (3.1)	65.5 (19.5)	68.8 (28.1)	
Clinical rank	General dentist	9.9 (3.0)	61.6 (19.0)	62.5 (31.3)	0.04
	Postgraduate resident	10.6 (1.9)	66.4 (11.9)	68.8 (18.8)	
	Specialist/consultant	10.5 (2.2)	65.4 (13.9)	62.5 (18.8)	

## Discussion

The Saudi MOH Manual of Infection Prevention and Control in Dental Settings provides a road map for healthcare sectors to establish and implement infection control policies based on the most updated and evidence-based practices. In Endodontics, the Saudi MOH policy primarily describes using and reprocessing of endodontic instruments such as endodontic files and barbed broach [3]. This study provides the first insight into the awareness, implementation, and compliance with the infection control policy for using and reprocessing endodontic files recommended by the Saudi MOH.

Previous studies in Saudi Arabia reported a high awareness level of the infection control standard precaution guidelines in dental clinics in Makkah (93.9%) and Hail (86%) [13,14]. However, this study showed that only 76.1% of the participants were aware of the manual recommended by the Saudi MOH, and only 71.1% used the manual as a guideline in their workplace. This variation in the reported awareness level between our study and previous studies may be due to differences in the sample selection criteria and the tested guidelines.

In endodontics, several studies have explored the knowledge and adherence to infection control guidelines during root canal treatment [15,16]. However, more data on adherence to the guidelines for reprocessing endodontic files nationally and internationally are needed. Sonntag et al. reported low compliance with the guidelines for reprocessing endodontic files in Germany [11]. In contrast, the results of this study showed moderate to high compliance of participants with the policy recommended by the Saudi MOH. This high level of compliance may be attributed to the efforts of healthcare sectors to meet the requirements of the Saudi Central Board for Accreditation of Healthcare Institutions, which has shown a positive impact on healthcare workers’ performance of infection control standard precautions [17].

In this study, experience level was the only demographic factor significantly affecting participants’ compliance with the policy. Participants with more than ten years of experience showed a higher level of compliance than those with less than 5 years of experience. This finding is consistent with that observed in several studies conducted in Jerusalem, Lebanon, and the Islamic Republic of Iran, which showed that more experienced clinicians showed better compliance with infection control policies [18-20]. This correlation may be due to the extensive exposure to training, mentorship, and supervision that they have received over the years [21].

Reusing endodontic files after sterilization in multiple patients has been a common practice for years. However, this practice is debatable due to several concerns, including the potential for prion cross-infection, the effect of sterilization on the durability and cutting efficiency of the files, the effect of multiple uses and instrument separation, and the possibility of corrosion due to exposure to different irrigation solutions [22]. From an infection control point of view, the decontamination and sterilization processes of different types of endodontic files have been reported to be inadequate, unreliable, and unpredictable, and pose a possible risk of transmitting infectious diseases [5-8]. Thus, some countries, such as the United Kingdom, have decided to consider endodontic files as single-use instruments [9]. In Saudi Arabia, the policy recommended by the MOH recommends using endodontic files as single-use instruments that can be reprocessed and used only on the same patient in case of multiple treatment visits and if the manufacturer’s instructions allow for such practice. Further, the policy recommends wiping the file with sodium hypochlorite after each insertion in the canal to remove any debris [3]. Nevertheless, only 13.2% of the participants in this study used endodontic files as single-use instruments, and only 36% of them wiped the files with sodium hypochlorite after canal preparation. Additionally, 83% of the participants discarded the barbed broach after a single use. These findings are similar to those reported in South Africa and the United States [23,24]. Furthermore, this study showed that 58.5% of the participants reused endodontic files 2-4 times. Logsdon et al. reported that 40.5% of clinicians discarded endodontic files after the third use [24]. The high cost of endodontic files may be attributed to this practice [25,26].

According to the Saudi MOH manual, endodontic files are categorized as "critical instruments," which require decontamination and sterilization before every use. Furthermore, it recommends reprocessing any instrument removed from the sterilization packaging, even if it was not used during the treatment. Moreover, the endodontic policy clearly states that it is prohibited to use a single endodontic box for multiple patients without sterilization [3]. However, 13.4% of the participants in this study did not reprocess the unused content of the endodontic box. A previous study in Jeddah showed that 54.8% of participants believed that removing one file from a sterile bag does not affect the sterility of the unused content of the bag [27]. This may be attributed to a lack of knowledge regarding the risk of bioaerosol contamination and the recommended policy.

Earlier studies have suggested that new, unused endodontic files are not sterile and should be sterilized before use [28-30]. A previous study in Jeddah revealed that 15.3% of clinicians sterilized new endodontic files before using them [27]. However, higher rates of sterilization of new endodontic files have been reported in the United States (61% of endodontists and 56% of general dentists) and Nigeria (50%) [31,32]. In this study, only 33.8% of clinicians sterilized new endodontic files. This practice may be due to a lack of knowledge and the absence of strict regulations.

Reprocessing used dental instruments is a common task for dental assistants. Therefore, their knowledge and practice of the sterilization process are of utmost importance for the safety of dental personnel and patients. Accordingly, several studies have investigated their knowledge and adherence to sterilization guidelines in endodontics. In Nigeria, dental assistants have been found to have a fair understanding of endodontic instrument sterilization [32]. In contrast, a lack of knowledge and deficiencies in training have been reported in Bangalore and Turkey [33,34]. In this study, approximately 61.7% of participants reported that reprocessing endodontic files is the responsibility of dental assistants, which is lower than the percentage reported in Germany [11]. However, the knowledge and compliance of dental assistants with the policy of sterilizing endodontic instruments in Saudi Arabia are currently unknown and require further investigation.

The Saudi MOH manual does not mandate the use of a washer disinfector to clean and disinfect used instruments before sterilization. However, some other countries require it to be used for all instruments classified as “critical instruments” before sterilization in the autoclave [35]. In this study, 61.9% of the participants used a thermal washer disinfector before autoclaving. This finding is consistent with a similar study conducted in Germany [11]. However, a study conducted in Glasgow showed that none of the surveyed clinicians used a washer disinfector [36].

This study provided new and valuable information about the common practice regarding the use and reprocessing of endodontic files and adherence to MOH policy in various healthcare sectors in Saudi Arabia. The sample size was adequate, and the questionnaire was validated. However, the study had some limitations. This was a self-administered questionnaire-based study. Thus, there may be a risk of selection bias. Although the overall sample size was adequate, the low sample size of some demographic groups might limit the ability to detect differences between the groups. Additionally, the results of this study do not necessarily generalize to different countries or healthcare systems that apply different guidelines. For future studies, we suggest using mixed methods and qualitative studies to look inwardly at the related concerns and challenges, get more specific data, and effectively plan for proper infection control training and development of action plans for implementation of the recommended guidelines. Also, more future studies may address the practice of reusing and reprocessing different types of endodontic instruments

## Conclusions

Our study shows strengths and areas of improvement regarding the awareness, implementation, and compliance of the use and reprocessing of endodontic files in Saudi Arabia. Although there is a relatively high level of compliance with the recommended policy, crucial measures such as the single-use of the endodontic files, sterilizing new endodontic files, and using the sterilized endodontic box for each patient need improvement. More efforts should be implemented toward improving awareness through continuous education and training to ensure better compliance with the infection control policy in endodontics, particularly among less experienced dentists.
